# Diversity of Planthoppers Associated with the Winter Rice Agroecosystems in Southern Yunnan, China

**DOI:** 10.1673/031.012.2901

**Published:** 2012-02-29

**Authors:** Shao-ji Hu, Da-ying Fu, Xiao-jun Liu, Tao Zhao, Zhong-liang Han, Jian-ping Lü, Hai-long Wan, Hui Ye

**Affiliations:** ^1^ Laboratory of Biological Invasion and Transboundary Ecosecurity, Yunnan University, Kunming, 650091, China; ^2^ Plant Protection and Quarantine Station of Yunnan Province, Kunming, 650034, China; ^3^ Southwest Forest University, Kunming, 650224, China

**Keywords:** Delphacidae, distribution, overwintering habitat, rice pests, Yunnan

## Abstract

A field survey of the overwintering planthoppers (Hemiptera: Delphacidae) associated with the rice agroecosystems in southern Yunnan was carried out during January-February in 2010 and 2011. 22 species of planthoppers were collected and identified, with one species representing the subfamily Stenocraninae and the other 21 species in Delphacinae. *Nycheuma cognatum* (Muir), *Peregrinus maidis* (Ashmead), and *Pseudosogata vatrenus* (Fennah) were new provincial records for Yunnan. The pest species, *Sogatella furcifera* (Horváth), *Nilaparvata lugens* (Stål), and *Laodelphax striatellus* (Fallén) were able to overwinter in part of the survey range. 13 species were listed to be of economic importance. Abandoned rice paddies with dense Poaceae grasses (Poaceae) were the most favorable overwintering habitat. The survey range was divided into four regions and five areas based on natural geographical characteristics. The study demonstrated that winter temperature differentiation, terrains, and habitat differences were three factors affecting planthopper diversity. Planthopper species diversity showed a reductive trend from south to north and reflected a gradient of more severe winter temperatures. In addition, planthopper diversity was influenced by smaller—scale differences in terrain and habitat, as evidenced by greater diversity in the valleys and low—altitude areas as compared to mid—mountain and Karst plain areas.

## Introduction

Planthoppers (Hemiptera: Delphacidae) are a large group of small herbivorous insects, most of which live on monocotyledons by feeding on phloem sap (Nault and Rodriguez 1985). Many planthoppers are serious pests on major agricultural crops worldwide (Wilson 2005; Dupo and Barrion 2009). Among them, *Nilaparvata lugens* (Stål), *Sogatella furcifera* (Horváth), and *Laodelphax striatellus* (Fallén), generally called rice planthoppers (Wu et al. 1987), are devastating pests of rice in tropical and temperate Asia (Dyck and Thomas 1979; Catindig et al. 2009; Cheng 2009).

China is one of the eastern Asian countries infested with rice planthoppers. Massive outbreaks of rice planthoppers occurred in the 1980s, and the problem is once again escalating and becoming a threat to rice production in China (Zeng and Xu 1992; Sogawa et al. 2003; Cheng 2009). Therefore, research on survey techniques and control strategies for rice planthoppers is a high priority for rice pest management in China (Cheng 2009).

Yunnan Province is the major rice—producing region in southwestern China, and is an area heavily infested with rice planthoppers (Fu et al. 2009). Yunnan has a complex terrain and climate, as well as high biodiversity (Wang and Zhang 2005), where 89 planthopper species have been officially recorded, 47 of which are grass—feeding species (Ding 2006). However, systematic planthopper surveys in rice agroecosystems, especially in winter rice agroecosystems, are extremely rare. To date, only the study by Yang et al. (1982) has been conducted in rice agroecosystems in Yunnan,where 12 species of overwintering planthoppers were reported.

During the past 20 years, cropping systems, rice varieties, and farmland management practices in Yunnan have changed significantly (YBS 1990, 2010), which could bring about changes in the species composition and distribution patterns of overwintering planthoppers in rice agroecosystems. This is an important concept, because the size of overwintering planthopper populations in rice agroecosystems is considered a key factor in predicting the outbreak potential in the subsequent year (Yang et al. 1982; Denno and Roderick 1990). Therefore, assessing the current status of overwintering planthoppers in rice agroecosystems should be an important component of surveillance programs for rice pests.

The aim of the present study was to clarify the current status of species composition, abundance, habitat affinities, and distribution patterns of overwintering planthoppers in the major rice growing regions of southern Yunnan, especially the economically important species, and to elucidate ecological characteristics of these planthoppers. The results of this study should allow for improved understanding of the outbreak dynamics of rice planthoppers in southern Yunnan and formulation of integrated pest management strategies for these insects.

## Materials and Methods

### Survey geographic range and field sites

**Figure 1.**
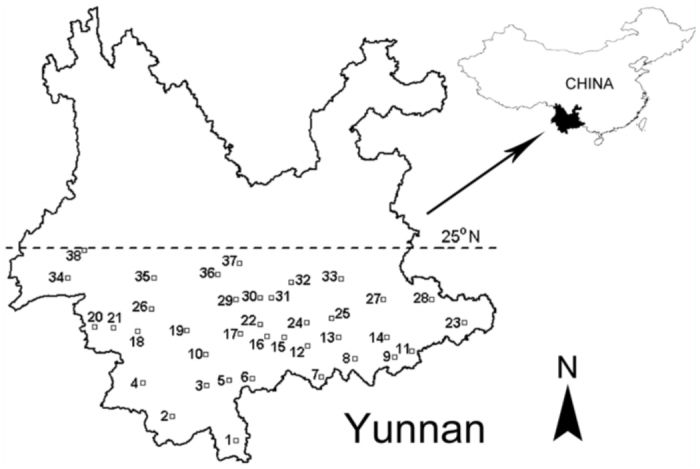
Geographic distribution of the 38 field sites surveyed in southern Yunnan. The numbers of field sites correspond to those given in Table 1. High quality figures are available online.

In an attempt to allow comparison with the previous research of Yang et al. (1982), the areas between the southern border of Yunnan up to 25 ^°^N latitude were set as the survey range. 38 field sites (Table 1, Figure 1) were selected within this area based on the following criteria: (1) localities had paddies presented year—round or in any other season apart from winter, (2) terrains such as plateaus, valleys, mountains, and hills were included, and (3) localities having previous reports of rice planthoppers infestation in spring and summer.

The winter rice agroecosystems in this study consisted of the paddies and adjacent non—agricultural lands covered with dense grass (Poaceae). Overall, six types of habitats were investigated: (1) paddies with actively growing rice, (2) paddies with ratooning rice, (3) abandoned paddies with dense grass, (4) stream and/or river banks with dense grass, (5) field drainages with dense grass, and (6) wheat plantations.

### Specimen collection and identification

Field surveys were carried out from January to February in both 2010 and 2011. Planthoppers were collected by sweep—netting at each study site. For each type of habitat at a field site, three 4 m^2^ sample areas were randomly selected (total sampling area was 1464 m^2^), and each sample area was swept 10 times with an insect net (net size: 30 cm in diameter, 85 cm in depth). Planthoppers were isolated from all the captured arthropods and preserved in 95% ethanol in 1.5 mL microtubes labeled with the site name and habitat type and then stored at room temperature. Identification to species was done in laboratory using a Nikon SMZ1500 stereoscope (www.nikon.com).

Only adult specimens were used in this study to ensure accurate identification. Male specimens were identified by the characteristics of the male genitalia. Due to the lack of viable characteristics of the female genitalia, female specimens were identified by morphological comparison with previously identified males or descriptions from Ding (2006). To observe the male genitalia, the apical portion of abdomen was treated with 10% NaOH solution and dissected in distilled water under the stereoscope. The checklist of planthoppers in the present study was arranged according to the classification and nomenclature proposed by Ding (2006). For each survey site, Microsoft Excel 2003 (www.microsoft.com) was used to summarize the number of identified species and number of specimens of each species. The data were also analyzed to look for patterns by habitat type and geographic region.

### Diversity and distribution

Based on the integrated natural regionalization system of Yunnan (Yang 1990) and environmental factors, such as mean ambient temperature in January and major agricultural cropping systems (He and Chen 1980; YPMB 1982), the survey range was divided into four regions and five areas. These regions and areas were: (I) Valley and low—altitudinal warm region, which consisted of three areas: (I_A_) southern border low—mountain area, (I_B_) southwestern mid—low—mountain area, and (I_c_) Red—River valley and altiplano area; (II) Pu'er mid—mountain strath region; (III) southeastern Karst plain region, which consisted of two areas: (III_A_) Wenshan Karst area and (III_B_) southern Nanpan—River area; (IV) Northern margin region. Species composition and abundance in each region and area was analyzed, and statistical results were mapped using ArcView 3.3 (ESRI, www.esri.com).

## Results

### Species composition

Overall, 441 adult planthopper specimens were collected during the field surveys and represented 22 species belonging to two subfamilies (one species of Stenocraninae and 21 species of Delphacinae, Table 2). These 22 species represent 46.8% of the known grass—feeding planthoppers in Yunnan (Ding 2006). Three species, *Nycheuma cognatum* (Muir), *Peregrinus maidis* (Ashmead), and *Pseudosogata vatrenus* (Fennah) were new records for Yunnan. Three rice planthoppers species, *L*. *striatellus*, *N*. *lugens*, and *S*. *furcifera* were recorded from several localities.

Among the 441 specimens, *Sogatella kolophon* (Kirkaldy) was the most common, with 92 specimens collected (20.9% of 441 specimens), followed by *S*. *furcifera* (80 specimens; 18.1%), *S*. *vibix* (Haupt) (71 specimens; 16.1%), and then *L*. *striatellus* (54 specimens; 12.3%). The remaining 18 species were only occasionally collected from the study sites (Table 2).

### Habitat affinities

**Figure 2.**
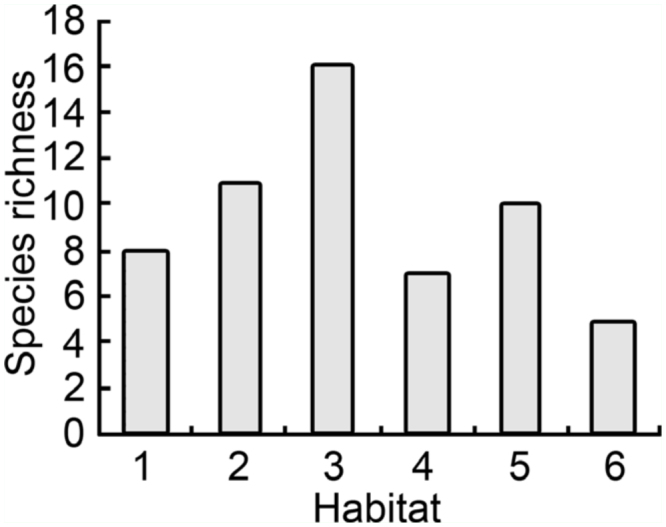
Number of species of planthoppers (total N = 22) collected in the six habitat types in southern Yunnan. The habitat types are described in Table 1. High quality figures are available online.

The habitat affinity analysis showed a clear selection preference for particular habitats by several species (Table 2, Figure 2). 16 species of planthoppers (72.7%) were collected from abandoned paddies with dense grasses, while only 5 species (22.7%) of planthoppers were found in the wheat plantation. 10 species (45.5%) of planthoppers were collected from field drainages with dense grass. Species abundance in paddies with ratooning rice was higher than in paddies with actively growing rice.

Apart from the general habitat preferences mentioned above, specific habitat preferences by certain groups of planthoppers were also noticed. For example, *L*. *striatellus*, *Ni*. *lugens*, *Ni*. *muiri* China, *Ni*. *bakeri* (Muir), *Ni*. *castanea* Huang et Ding, *S*. *furcifera*, *S*. *vibix*, *S*. *kolophon*, and *Tagosodes pusanus* (Distant) were found primarily in habitats closely associated with paddies, such as paddies with ratooning rice, abandoned paddies with dense grass, field drainages with dense grass, and wheat plantations (Table 2).

### Diversity and distribution

**Figure 3.**
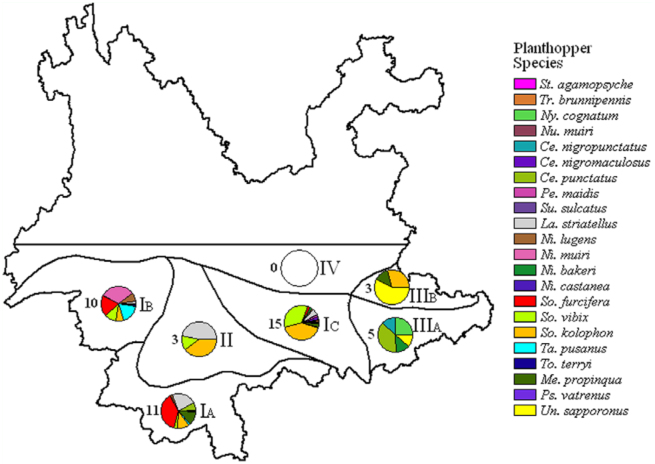
Diversity and distribution mapping of the 22 species of planthoppers collected from winter rice agroecosystems in southern Yunnan with the species amounts on the left side of the pie charts and the region/area labels on the right side. High quality figures are available online.

Twenty species (90.9%) of planthoppers were collected from region I with 11 species from area I_A_, 10 species from area I_B_, and 15 species from area I_c_. Three species (13.6%) of planthoppers were collected from region II. Overall, seven species (31.8%) were captured from region III with five species (22.7%) from area III_A_ and three species (13.6%) from area III_B_. NO planthoppers were found in region IV during the surveys (Table 3, Figure 3).

The distribution of some planthopper species appeared restricted to certain areas (Figure 3). *Nilaparvata muiri*, *S*. *furcifera*, *T*. *pusanus*, and *P*. *vatrenus* were primarily found in the valleys and low—altitudinal areas in southwestern Yunnan (areas I_A_ and I_B_). *Stenocranus agamopsyche* Kirkaldy, *Tropidocephala brunnipennis* Signoret, *Cemus nigromaculosus* (Muir), *P*. *maidis*, and *Sulculus sulcatus* Ding were captured only from the Red—River valley and altiplano area (area I_c_). *Nycheuma cognatum*, *C*. *nigropunctatus* (Matsumura), *C*. *punctatus* (Muir), and *Unkanodes sapporonus* (Matsumura) were primarily collected in the southeastern Karst plain region (area III_A_ and III_B_). By contrast, planthoppers such as *L*. *striatellus*, *S*. *vibix*, and *S*. *kolophon* were commonly found across the entire survey range.

## Discussion

### Species and economic importance

In comparison to previous research by Yang et al. (1982), five species, *Harmalia sameshimai* (= *Delphacodes sameshimai*), *Terthron albovittata*, *Perkinsiella saccharicida*, *Harmalia sirokata* (= *Sogata sirokata*), and *Tagosodes baina* (= *Himeunka baina*) were not found in the present study; however, 16 previously unreported species were recorded in the present study. In recent years, multicropping agricultural systems have been promoted in much of southern Yunnan, which decreases the number of fallow fields in winter (Zhang et al. 2000), and consequently may affect the overwintering habitats of many planthoppers. The differences in species abundance between the present study and the previous study (Yang et al. 1982) may have resulted primarily from the changes in cropping systems and land utilization in winter. Moreover, some species of planthoppers may overwinter as eggs or nymphs (Denno and Roderick 1990), which are difficult to collect, and this could be another possible cause of such difference in species composition. Future bionomic studies of these planthoppers would provide more information.

The white—backed planthopper, *S*. *furcifera*, a severe rice pest in Yunnan, was primarily found in habitats closely related to rice paddies. The overwintering *S*. *furcifera* are believed to be responsible for any outbreak that occurs in the subsequent year (Liu et al. 1991). However, the extremely small population size of overwintering *S*. *furcifera* during our survey can hardly explain the outbreak that occurred in late spring and early summer of 2010 and 2011. The surveys conducted during the present study were adjacent to the northern portion of the Indochinese peninsula, where *S*. *furcifera* is able to maintain sizable populations in winter (Peter A.C. Ooi personal communication). Therefore, the authors believe that the overwintering populations of *S*. *furcifera* in southern Yunnan are only partly responsible for any future outbreaks that occur there in southern Yunnan. Detailed overwintering areas for *S*. *furcifera* in Yunnan as well as the source and composition of its erupting populations will be discussed in a separate paper.

The following 12 planthopper species were closely associated with rice agroecosystems: *Ny*. *cognatum*, *Numata muiri* (Kirkaldy), *P*. *maidis*, *L*. *stratellus*, *Ni*. *lugens*, *Ni*. *bakeri*, *Ni*. *muiri*, *S*. *vibix*, *S*. *kolophon*, *T*. *pusanus*, *Metadelphax propinqua* (Fieber), and *U*. *sapporonus*. Previous research demonstrated that these planthoppers are key vectors of some serious rice phytopathogens (Nault and Ammar 1989; Wilson 2005; Liu et al. 2010), which have become another major threat to intensive rice cultivation in recent years. Hence, surveillance for these planthoppers in the rice producing areas of southern Yunnan should be taken into consideration in the future.

### Diversity distribution and effecting factors

Yunnan is a region of China with very high biodiversity (Guo and Long 1998). The spatial distribution pattern of tephritid pests in Yunnan suggested that mountainous terrain, altitudinal differentiation, and habitat diversity were the principal causes for high species abundance compared to other areas in China located at the same latitude (Chen et al. 2010).

The planthopper species abundance generally decreases in a northward direction in Yunnan and is also influenced by small—scale altitude and temperature changes. The mean winter temperature (i.e., the mean January temperature) in Yunnan decreases with the increasing latitude (Chen 2001), and similarly the planthopper diversity decreases with increasing severity of winter temperature.

The southern and southwestern portions of Yunnan are located south of the longitudinal range—gorge region in Yunnan, with the mountain ranges to the north preventing in large part the southerly movement of cold winter air currents (Chen 2001). For example, the mean January temperatures in areas I_A_ (northern tropical) and I_B_ (southern subtropical) are ∼ 12–15 ^°^C and ∼ 9–12 ^°^C, respectively (YPMB 1982; Wang and Zhang 2005), and planthopper diversity was highest in these two areas. By contrast, planthopper diversity was much lower in the southeastern portion of Yunnan (region III, mean January temperature ∼ 6–9 ^°^C, climatic type mostly central subtropical) is situated in the western margin of the Yunnan—Guizhou plateau, which is much more exposed to the cold winter air current (YPMB 1982; Xie and Yang 1995; Wang and Zhang 2005; He and Zhang 2007).

Small—scale terrain is another factor affecting the diversity distribution of planthoppers. Four species of planthoppers recorded in this study were mostly found in region I in southern and southwestern portions of Yunnan, while five other species were captured only in the Red—River valley. These two areas are located in the southern part of the longitudinal range—gorge region of Yunnan, where major mountains and rivers run parallel (Wang and Zhang 2005). The combination of longitudinally connected, horizontally obstructed, fragmented, and isolated habitats (He et al. 2005) may restrict the distribution of some species and lead to the distinct distribution pattern observed in this study.

Habitat differences also influenced the diversity and distribution of planthoppers. 16 species of planthoppers were recorded in the abandoned paddies with dense grass, whereas only five species were found in wheat plantations. Grasses such as *Oryza* spp., *Echinochloa* spp., *Paspalum* spp., and *Leersia* spp. in the abandoned paddies serve as overwintering food hosts of many planthoppers (Lei et al. 1983; Ma 1985; Denno and Perfect 1994; Ding 2006; Yu et al. 2009). In comparison, planthopper species abundance in paddies with ratooning rice (mostly *O*. *sativa*) was much lower, probably reflecting the presence of monoculture vegetation. Similarly, species abundance of planthoppers in paddies with actively growing rice or wheat plantations was much lower, again possibly reflecting intense monoculture and frequent disturbance. Notably, 10 species of planthoppers were recorded from the field drainages with dense grass, which may reflect the habitat heterogeneity of the drainages and as well as edge effects (Harris 1988).

**Table 1.**
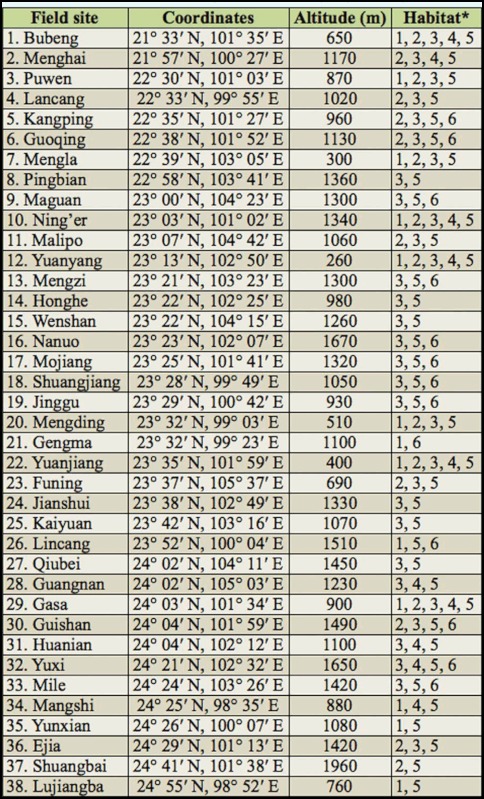
Summary information for the 38 field sites surveyed in Yunnan arranged by ascending order of latitude.

**Table 2.**
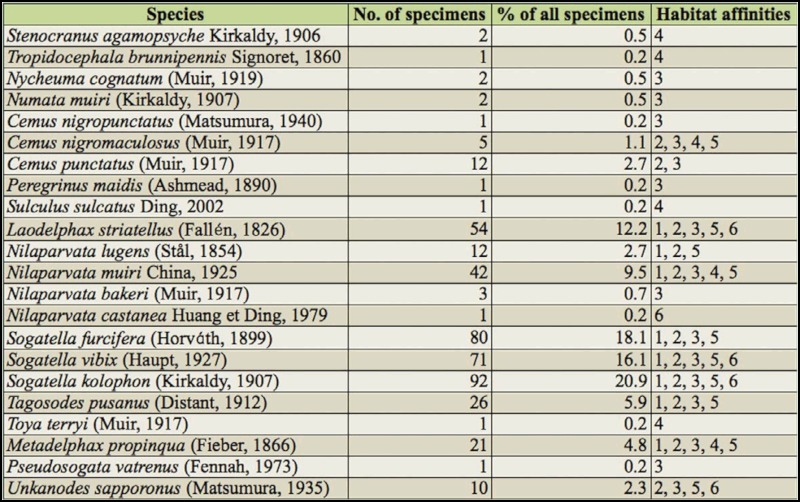
Checklist of the 22 species of planthoppers collected from winter rice agroecosystems in southern Yunnan with the number of specimens collected and their primary habitat affinities. The numbers of habitat types correspond to those given in Table 1.

**Table 3.**
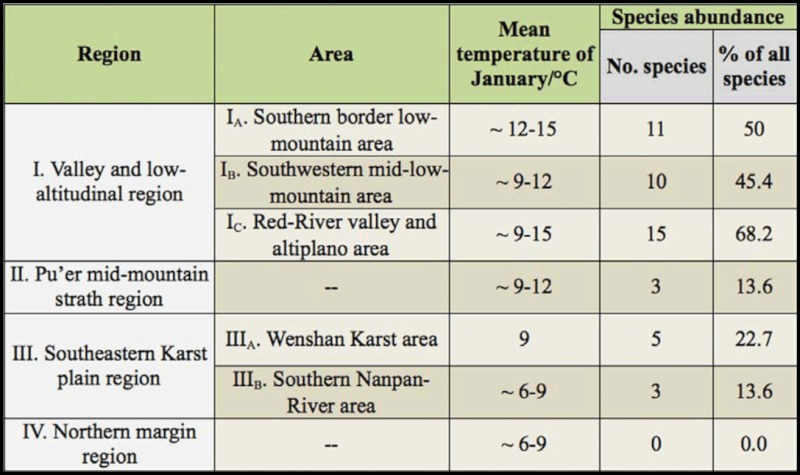
Species abundance of the 22 species of planthoppers collected in southern Yunnan by geographic region and area as well as the range of mean January temperatures for each region and area.
